# Occurrence of Deoxynivalenol and Zearalenone in Commercial Fish Feed: An Initial Study

**DOI:** 10.3390/toxins5010184

**Published:** 2013-01-16

**Authors:** Constanze Pietsch, Susanne Kersten, Patricia Burkhardt-Holm, Hana Valenta, Sven Dänicke

**Affiliations:** 1 Programm Mensch-Gesellschaft-Umwelt, Department of Environmental Sciences, University of Basel, Vesalgasse 1, Basel CH-4051, Switzerland; E-Mail: patricia.holm@unibas.ch; 2 Friedrich-Loeffler-Institute (FLI), Federal Research Institute for Animal Health, Institute of Animal Nutrition, Bundesallee 50, Braunschweig D-38116, Germany; E-Mails: susanne.kersten@fli.bund.de (S.K.); hana.valenta@fli.bund.de (H.V.); sven.daenicke@fli.bund.de (S.D.)

**Keywords:** *Fusarium* toxins, aquaculture, feedstuffs

## Abstract

The control of mycotoxins is a global challenge not only in human consumption but also in nutrition of farm animals including aquatic species. *Fusarium* toxins, such as deoxynivalenol (DON) and zearalenone (ZEN), are common contaminants of animal feed but no study reported the occurrence of both mycotoxins in fish feed so far. Here, we report for the first time the occurrence of DON and ZEN in samples of commercial fish feed designed for nutrition of cyprinids collected from central Europe. A maximal DON concentration of 825 μg kg^−1^ feed was found in one feed whereas average values of 289 μg kg^−1^ feed were noted. ZEN was the more prevalent mycotoxin but the concentrations were lower showing an average level of 67.9 μg kg^−1^ feed.

## 1. Introduction

The mycotoxins deoxynivalenol (DON) and zearalenone (ZEN) appear due to cultivation of cereals world-wide due to their production as secondary metabolites by naturally occurring fungi of the genus *Fusarium* [1]. Introduction of these mycotoxins into soil takes places by contaminated parts of the cereals, and subsequently these substances can be transferred to aquatic environments [2]. Due to their relative high stability in the aquatic environment, they can be found in relevant concentrations in surface waters [2,3]. Even more importantly, fish in aquaculture are commonly exposed to feed-borne mycotoxins. Commercial feed for aquaculture often contain high amounts of fishmeal [4], but the growing aquaculture decreased the fish meal stock world-wide and the decreasing availability of fish resources led to the need for alternative protein resources to replace fishmeal in these feed. Thus, cereals are increasingly used for production of fish feed [5].

DON contamination in animal feed components and finished feedstuff is very prevalent in Europe and North America [6]. However, it has up to now only rarely been shown that DON and ZEN are contaminants of fish feed components [7,8], but wheat and less frequently corn, barley, and rye are used for feed production and might contain these mycotoxins in relevant concentrations with high prevalency [9,10,11]. Especially, wheat is often used for fish feed production due to its high protein content and its benefits concerning the preservation of the pellet shape during the production process of pelleted feed. The amount of wheat in fish feed varies considerably, ranging from approximately 15 to 27 percent for carnivorous fish whereas feed for cyprinids usually contain 20 to 70 percent [11,12]. In addition, whole cereals can also be used as supplementary feed in semi-intensive culture of fish species such as carp [13]. 

The undesired introduction of cereal-borne mycotoxins into fish feeds leads to so far largely unknown consequences. In mammalian cell lines, it has been found that ZEN is hepatotoxic [14,15], immunotoxic [16,17], and genotoxic [18]. The toxicity of DON is also well recognized in mammalian cell lines including mechanisms such as mitochondrial impairment and apoptosis [19,20], but few studies have assessed its toxicity on cell lines of aquatic organisms [21,22]. 

Recent investigations on salmonids showed that alterations in the intestinal tract of fish occur upon feeding with DON-contaminated diets [23,24]. A study on zebrafish (*Danio rerio*) showed effects of DON on fecundity and offspring larvae swimming activity [25]. ZEN and its metabolites, namely α-zearalenol (α-ZEL) and β-zearalenol (β-ZEL), have been shown to act as typical estrogenic compounds with partially high estrogenic potencies via affinity to estrogen receptors of fish depending on the fish species [26,27]. Thus, investigations on effects of ZEN in fish focussed on reproduction of zebrafish and early life stages of fathead minnow (*Pimephales promelas*) [28,29]. Only one study investigated the effects of ZEN on growth performance of Atlantic salmon (*Salmo salar*) showing no evidence for an adverse influence of ZEN on this species at concentrations ranging from 60 to 770 μg kg^−1^ feed [23].

Since the sensitivity of farm animals to DON differs remarkably, different guideline values of DON in feed have been proposed [30]. A general recommendation on guidance values of 5 mg kg^−1^ DON in complete feedingstuff was established by the European Commission [31] while specific recommendations concerning ZEN contamination in animal feed are only referring to some animal species such as pigs, cattle, sheep and goats. Nevertheless, DON concentrations below its guidance value have already been shown to affect fish negatively [23,24,25].

Still the occurrence of *Fusarium* toxins in aquaculture remains mostly unknown and research is needed. Here, we present DON and ZEN concentrations in commercial feed mainly designed for cultivation of cyprinids in order to assess their possible health risks for aquatic animals and to provide a first basis for deriving acceptable guidance values for DON and ZEN in fish feed.

## 2. Results and Discussion

### 2.1. Occurrence of DON

The study of Måge *et al*. [7] reported a contamination prevalence with DON in fish feed components of 33%, whereas others reported 50% DON contamination in samples from cereals, straw, silage and finished feed from all over the world [32]. In contrast, we were able to show that DON could be found in more than 80% of the samples from commercial fish feed showing average values of 289 μg kg^−1^ feed (Table 1). A maximum value for DON of 825 μg kg^−1^ was noted for feed #6. However, its metabolite DOM-1 was not found in the samples. Thus, the DON values in fish feed do not exceed the guidance values for complete feedingstuff [31].

### 2.2. Occurrence of ZEN

Again Måge *et al*. [7] noted that 33% of the samples taken from fish feed components were contaminated with ZEN, whereas another study reported that in 56% of the samples from finished animal feed ZEN was found with average levels of 99 μg kg^−1^ feed [6]. In Asian countries ZEN was found in 9 fish feed samples showing average concentrations of 76.2 μg kg^−1^ feed [8]. Similarly, in our study ZEN was found in all samples (Table 1), although the concentrations varied considerably (average 67.9 μg kg^−1^ feed, ranging from 3 to 511 μg kg^−1^ feed). Consequently, the ZEN values found in our study do not exceed the values currently recommended by the European Commission [31].

**Table 1:** Deoxynivalenol (DON) and zearalenone (ZEN) concentrations in commercial fish feed in central Europe designated for feeding of cyprinids.

Feed	Pellet size (mm)	Dry matter (%)	Crude protein ^1^ (%)	Crude fat ^1^ (%)	DON (μg kg^−1^)	ZEN (μg kg^−1^)
#1	2.0	90.9	20.0	4.8	768	80
#2	4.5	86.9	42.2	23.8	81	10
#3	1.6	90.7	48.6	13.1	284	15
#4	4.5	92.0	48.6	13.1	117	27
#5	2.8	91.0	48.6	13.1	66	9
#6	3.0	92.2	33.0	6.0	825	511
#7	3.0	94.5	30.0	5.0	150	8
#8	3.0	92.3	34.0	15.0	0	6
#9	3.0	92.5	45.0	12.0	0	3
#10	2.5	91.9	41.0	12.0	176	12
#11	3.0	91.6	35.0	6.0	131	21

^1 ^According to the manufacturer.

### 2.3. Possible Consequences of DON and ZEN Contamination in Fish Feed

According to the recommendation of the European Commission [31] ZEN should not exeed levels of 2 mg kg^−1^ feed material (with the exception of maize by-products) and DON should be lower than 5 mg kg^−1^ complete feedstuff. According to our findings for the samples from fish feed these values are not exeeded. However, recent studies showed that DON can be harmful to fishes at levels below these recommendations [23,24,25]. For ZEN the consequences of food-borne exposure remain mostly unknown [23]. Still, the frequent exposure of fish to single mycotoxins and combinations of different mycotoxins in aquaculture represents a continuous health risk [29,33,34]. Unfortunately, the actual risks remain more or less unknown due to a lack of data which provide evidence for effects in aquatic animals and which clarify the mechanism(s) of action of these substances. In particular, the effects of DON on certain aspects of fish health and cell function *in vivo* have rarely been investigated in fish [24]. However, this is an important issue for future research since economic consequences for the animal industry can be minimized.

### 2.4. Possible Strategies to Prevent DON and ZEN Contamination in Fish Feed

Chemical substances and natural toxins in the food chain received increasing attention recently which led to risk assessments and the development of different prevention strategies [35,36]. One strategy would include the selection of proper ingredients for feed production. Not surprisingly, even within one production firm the batches of ingredients seem to influence the mycotoxin content of the final feed enormously. This can be seen from feed #3 to #5 which were produced by the same company by using similar ingredients and the same production processes but differed in final pellet size. Nevertheless, the contents of DON and ZEN differ considerably between these three samples (Table 1). Moreover, the contamination of the tested fish feed samples in our study was more prevalent than previously expected from other studies [6,7]. This is certainly due to the combination of ingredients that were chosen for feed production for cyprinids. A quite compatible protein source in fish feed would be reached by inclusion of fish meal at high percentages. However, the availability of fish meal for production of aquaculture feeds is decreasing globally over the last decades and the fish meal prices are increasing accordingly [5]. Thus, alternative protein sources need to be used for fish feed production. A higher percentage of cereals is commonly used for feed production for cyprinids compared to carnivorous fishes [11,12]. But natural contamination of commercial fish feed with DON and ZEN seems to be higher when corn and cereal components are used at higher percentages whereas fish meal-based feed mostly contained less of these mycotoxins (Table 1 and Table 2). This assumption is supported by the finding that the most prevalent mycotoxin in central Europe was DON with maximum values of 14.1 mg kg^−1^ barley and 49.0 mg kg^−1^ wheat (found in Austria) and average levels of ZEN and DON of 1.8 mg kg^−1^ and 1.7 mg kg^−1^ in corn gluten meal world-wide, respectively [6]. Soybean and soybean meal are considered to be less contaminated with DON and ZEN [6,37] and the usage of these plant components instead of cereals probably leads to less contamination of animal feedstuffs with *Fusarium* toxins. However, soybean and soybean by-products can be used for fish feed production only at relatively low percentages (depending on the fish species) because they exert estrogenic effects and negative impacts on fish growth at higher levels [38,39]. Thus, the contribution of certain ingredients to the mycotoxin concentrations in fish feed and their possible consequences on fish health should be the subject of future research.

**Table 2:** Approximate declaration of ingredients of the commercial fish feed from central Europe; all components (C1 to C10) are sorted by decreasing percentage in the final feed; wheat, wheat by-products and rye are shaded in blue; fish components are displayed in green, blood meal is shaded in red, ingredients containing soybean are displayed in orange, and corn and corn by-products are shaded in yellow.

Feed	C1	C2	C3	C4	C5	C6	C7	C8	C9	C10
#1	W	WB	MS	CGF	SFEM	SEM	B	SBM	VO	M
#2	FM	FO	BM	SEM	WDB	W	M	Y	WB	VO
#3	FM	BM	W	SM	FO	M	-	-	-	-
#4	FM	BM	W	SM	FO	M	-	-	-	-
#5	FM	BM	W	SM	FO	M	-	-	-	-
#6	SEM	W	FM	WB	C	CGF	SFEM	FO	WGF	-
#7	SEM	WB	W	SFEM	FO	FM	-	-	-	-
#8	FM	W	SEM	FO	BM	VO	-	-	-	-
#9	FM	W	SEM	WGF	FO	VO	-	-	-	-
#10	W (31%)	SEM (27%)	FM (19%)	BM (6%)	FO (3%)	VO (3%)	-	-	-	-
#11	SEM	W	SM	FM	FO	M	-	-	-	-

**Abbreviations: **B = barley; BM = blood meal; C = corn; CGF = Corn gluten feed; FM = fish meal; FO = fish oil; M = minerals; MS = malt sprouts; SBM = sugar beet molasses; SEM = soybean extraction meal; SM = soybean meal; SFEM = sunflower feed extraction meal; VO = vegetable oils and fats; W = wheat; WB = wheat bran, WDB = wheat distillery by-product; WGF = wheat gluten feed; Y = yeast.

However, a general problem during our collection of samples was that although the producers of fish feed have to give an ingredient declaration of compounds in feedingstuffs by (percentage) weight of inclusion, only the producer of feed #10 displayed percentages on the packaging. All others refused to declare the percentages even on repeated enquiry. Therefore, an improved labeling policy would help to identify and prevent sources of mycotoxin inclusion in animal feed. 

It is also known that the storage of ingredients and the production processes influence mycotoxin contents in fish feed. Pelleted feed are increasingly replaced by extruded diets. Extrusion of feeds increases the digestibility of nutrients. The process of extrusion comprises an increase of temperatures to 110–160 °C. In our study feed #7 and feed #9 are extruded but, both of them still contain ZEN and feed #7 still contains DON. Studies on effects of high temperatures and high pressure on DON concentrations during production of food and feed have yielded contradicting results [40,41,42]. However, extrusion cooking clearly led to the reduction of DON concentrations in wheat grits depending on the adjustment of physicochemical parameters [43]. Moreover, extrusion can influence the content of ZEN [44]. We have no proof whether the ZEN content in samples from feed #7 and feed #9 was influenced by the production process. Nevertheless, according to the literature the selection of the appropriate course of production processes in addition to optimized storage conditions of ingredients and finished feed should lead to less mycotoxins in feedstuffs to some extent. 

In addition, some research group focus on strategies of biological detoxification of mycotoxins, the use of different sorbents or the use of genetically-modified crops for feed production in order to achieve less mycotoxin contamination in feed [45,46,47].

## 3. Experimental Section

### 3.1. Fish Feed Samples

For analyses of DON and ZEN 11 samples from fish feed from central Europe were collected. The samples that were chosen were mainly designed for use of carp feeding, since cyprinids display a major group of species in freshwater aquaculture [5]. All samples were taken from complete feedstuffs with the exception of feed #1 which is designated as a complementary feed for carp. Samples were stored at −20 °C before extraction of mycotoxins. 

### 3.2. Mycotoxin Analyses

DON and DOM-1 in fish feed were analyzed by HPLC-DAD (high performance liquid chromatography (consisting of a pump (LC-10ADVP), an autoinjector (SIL-10ADVP), and a column oven (CTO-10ACVP)) from Shimadzu (Duisburg, Germany)) with diode array detection using the detector SPD-M10AVP (Shimadzu, Duisburg, Germany) after a clean-up with IAC (immuno-affinity columns, DONprep^TM^, R-Biopharm, Darmstadt, Germany) according to manufacturer’s procedure with slight modifications as described previously [48]. The detection limit was 30 μg kg^−1^, the mean recovery was approximately 90%. 

ZEN in feed was determined by HPLC with fluorescence detection after a clean-up with IAC (ZearalaTest, Vicam, Klaus Ruttmann, Hamburg, Germany) according to a slightly modified method of the “Verband Deutscher Landwirtschaftlicher Untersuchungs- und Forschungsanstalten” [49]. The detection limit was 2 μg kg^−1^ and mean recovery was approximately 79%.

## 4. Conclusions

The values of DON and ZEN in commercial fish feed appear to be lower than the currently recommended values [31]. Still, it can be concluded that some of the commercial feed that were investigated within the present study may be harmful to fish due to their DON content. Unfortunately, we were only able to analyse 11 different fish feed so far. A much broader survey should be conducted to increase knowledge on the occurrence of cereal-borne toxins in feed designated for aquatic species. In this respect it can also be assumed that fumonisins and ergot alkaloids certainly also occur in fish feed but no survey has as yet assessed their concentrations in feed produced for aquatic species.
